# Combined RNAi-Mediated Suppression of Rictor and EGFR Resulted in Complete Tumor Regression in an Orthotopic Glioblastoma Tumor Model

**DOI:** 10.1371/journal.pone.0059597

**Published:** 2013-03-15

**Authors:** Maite Verreault, Sherry A. Weppler, Amelia Stegeman, Corinna Warburton, Dita Strutt, Dana Masin, Marcel B. Bally

**Affiliations:** 1 Experimental Neurooncology, Brain and Bone Marrow Institute Research Center, Pitie-Salpetriere Hospital, Paris, France; 2 Experimental Therapeutics, British Columbia Cancer Agency, Vancouver, BC, Canada; 3 Faculty of Pharmaceutical Sciences, University of British Columbia, Vancouver BC, Canada; 4 Department of Pathology and Laboratory Medicine, University of British Columbia, Vancouver BC, Canada; 5 Center for Drug Research and Development, Vancouver, BC, Canada; Bauer Research Foundation, United States of America

## Abstract

The PI3K/AKT/mTOR pathway is commonly over activated in glioblastoma (GBM), and Rictor was shown to be an important regulator downstream of this pathway. EGFR overexpression is also frequently found in GBM tumors, and both EGFR and Rictor are associated with increased proliferation, invasion, metastasis and poor prognosis. This research evaluated *in vitro* and *in vivo* whether the combined silencing of EGFR and Rictor would result in therapeutic benefits. The therapeutic potential of targeting these proteins in combination with conventional agents with proven activity in GBM patients was also assessed. *In vitro* validation studies were carried out using siRNA-based gene silencing methods in a panel of three commercially available human GBM cell lines, including two PTEN mutant lines (U251MG and U118MG) and one PTEN-wild type line (LN229). The impact of EGFR and/or Rictor silencing on cell migration and sensitivity to chemotherapeutic drugs *in vitro* was determined. *In vivo* validation of these studies was focused on EGFR and/or Rictor silencing achieved using doxycycline-inducible shRNA-expressing U251MG cells implanted orthotopically in Rag2M mice brains. Target silencing, tumor size and tumor cell proliferation were assessed by quantification of immunohistofluorescence-stained markers. siRNA-mediated silencing of EGFR and Rictor reduced U251MG cell migration and increased sensitivity of the cells to irinotecan, temozolomide and vincristine. In LN229, co-silencing of EGFR and Rictor resulted in reduced cell migration, and increased sensitivity to vincristine and temozolomide. In U118MG, silencing of Rictor alone was sufficient to increase this line’s sensitivity to vincristine and temozolomide. *In vivo*, while the silencing of EGFR or Rictor alone had no significant effect on U251MG tumor growth, silencing of EGFR and Rictor together resulted in a complete eradication of tumors. These data suggest that the combined silencing of EGFR and Rictor should be an effective means of treating GBM.

## Introduction

The median survival for patients with glioblastoma (GBM) is 41 weeks and the 2-year survival rate is less than 30% [1]. This poor survival rate is associated with the fact that malignant glioma is a highly aggressive and infiltrative disease typically resistant to standard chemotherapeutic agents [2]. Recurrence after treatment is still nearly universal [2] and it is now becoming obvious that conventional treatment approaches (surgery, radiation and/or chemotherapy) for these patients must change if improved treatment outcomes are going to be achieved. Although it is recognized that advances in GBM treatment will continue to rely on current treatment modalities, agents targeting proteins or pathways known to be critical to the progression and infiltration of GBM cells are now being evaluated in patients. These agents, used alone and in combination with established treatments, will hopefully improve treatment outcomes for individuals diagnosed with this devastating cancer.

The design of targeted therapy for achieving optimal therapeutic effects is complex. Cancer cell proliferation, growth and death are regulated by intricate networks of signaling pathways, and it is very likely that inhibiting any one specific pathway will activate compensating mechanisms. It is expected that the full potential of targeted therapy will only be realized by targeting multiple biological pathways in order to effectively impede cancer progression and recurrence [3]. Several important factors must be considered in the design of combination treatments involving agents targeting multiple pathways. First, the targeted therapeutic agents used cannot adversely affect outcomes achieved with existing treatments (e.g. radiation therapy or conventional cytotoxic agents). Second, subgroups of patients who are most likely to benefit from silencing of the targeted proteins or pathways must be defined through comprehensive pre-clinical and clinical studies. Third, targeted therapeutics must be used in combination settings in order to prevent the activation of compensating mechanisms in tumors. Finally, this approach will only be valuable for the treatment of GBM if the targeted agents are highly selective and capable of crossing the blood-brain barrier. In this study, the therapeutic potential of simultaneous silencing of the epidermal growth factor receptor (EGFR) and the rapamycin-insensitive companion of mTOR (Rictor) was assessed *in vitro* and *in vivo* and the rationale for selecting these proteins as therapeutic targets has been outlined below.

One of the most commonly reported molecular defects in GBM is the phosphatase and tensin homolog (PTEN), a negative regulator of the PI3K/AKT pathway. PTEN is mutated in 25–60% of GBM tumors [4], [5] and constitutive activation of the PI3K/AKT pathway, due to PTEN mutation, is associated with increased proliferation rate, invasion, metastasis and poor prognosis [6]–[8]. Moreover, Molina et al. [9] recently demonstrated, using *in vivo* orthotopic models of GBM, a strong correlation between AKT activation and GBM growth rate and invasiveness. Thus, tremendous efforts have been made to define strategies that inhibit the aberrant PI3K/AKT signaling for treatment of GBM (e.g. inhibitors of PI3K, AKT, PDK1, mTOR) [10]. The activation of AKT through phosphorylation is known to activate mTOR (mammalian target of rapamycin), which regulates a variety of functions associated with tumor pathogenesis [11], [12]. mTOR functions in two distinct multi-component protein complexes, both of which can influence AKT signaling. Inhibition of mTOR Complex 1 (mTORC1) can activate AKT, an effect attributed to Ribosomal S6 Kinase 1 (S6K1) -mediated feedback mechanisms [11], [13]–[16]. Alternatively, it was recently demonstrated that mTOR Complex 2 (mTORC2) can activate AKT through direct phosphorylation at its serine 473 site (p(ser473)AKT) [17], [18]. All known mTORC2 functions require the presence of the protein Rictor [19] and silencing of Rictor was reported to decrease p(ser473)AKT in GBM cells [20]. This latter study also reported elevated levels of Rictor protein in human GBM tumor tissue and cell lines when compared to normal brain tissue [20].

Epidermal Growth Factor Receptor (EGFR) overexpression or overactivation is also commonly observed in GBM tumors (40–70% of the patients) [21]–[23]. EGFR overexpression has been correlated with treatment resistance [24], as well as poor survival and poor prognosis [25]. Further, it has been demonstrated that the expression of a specific mutant form of EGFR (EGFRvIII) promotes tumor formation and growth (reviewed in [26]). The oncogenic properties of EGFRvIII overexpression are believed to be a consequence of the constitutive activation of downstream pathways such as PI3K/AKT [27]. This mutant form of EGFR lacks the Endothelial Growth Factor (EGF) binding site, thereby exhibiting a reduced internalization rate and promoting continuous signaling in the absence of growth factors [28]. The EGFR pathway, including downstream signaling proteins such as src and Ras/MAPK, is therefore considered by many as an appropriate therapeutic target in GBM [25], [29]–[33].

It is suggested here that Rictor silencing strategies, when combined with EGFR silencing, will result in optimal therapeutic effects in GBM. RNA interference (RNAi) methods were used to study the effects of combined silencing of Rictor and EGFR. An *in vitro* assessment of the approach was done using siRNA transfection in a panel of three EGFR overexpressing GBM lines, including two PTEN mutant lines (U251MG and U118MG) and one PTEN-wild type line (LN229). The results suggest that siRNA mediated co-silencing of EGFR and Rictor inhibits tumor cell migration in U251MG and LN229. In all three lines, the combined silencing strategy increased sensitivity to conventional chemotherapeutic agents known to be active in patients with GBM. *In vivo* validation of the co-targeting strategy was done using doxycycline-inducible shRNA-expressing GBM lines implanted orthotopically. The results demonstrate that silencing of EGFR or Rictor alone had no significant effect on tumor growth in the orthotopic U251MG GBM model, but the dual silencing of EGFR and Rictor *in vivo* results in eradication of the tumor.

## Materials and Methods

### Cell Culture and siRNA Transfection

U251MG, U118MG, LN229 glioblastoma and 293T embryonic cell lines were purchased from American Type Culture Collection (Manassas, VA). The mycoplasma status of these cells was confirmed negative by PCR (performed by Idexx Radil Laboratories, Columbia, MO). GBM4 and Gli36 were a gift from Dr Hiro Wakimoto from the MGH Molecular Neurosurgery Laboratory. U251MG, U118MG, LN229 and Gli36 cell lines were maintained in DMEM medium supplemented with 1% L-glutamine, 1% penicillin/streptomycin (DMEM, L-glutamine and penicillin/streptomycin from Stem Cell Technologies, Vancouver, British Columbia, Canada) and 10% fetal bovine serum (Hyclone, Logan, UT). GBM4 cell line was maintained in ultralow attachment plates (Stem Cell technologies) with Neurocult NS-A basal medium supplemented with Neurocult proliferation supplement (Stem Cell technologies), epidermal growth factor (20 ng/ml), fibroblast growth factor (10 ng/ml) (Clonetics®; Lonza, Walkersville, MA, USA) and heparin (2 µg/ml; Pharmaceutical Partners of Canada, Richmond Hill, ON, Canada). U251MG, U118MG and LN229 cells were transiently transfected with EGFR or Rictor siRNA, alone or in combination, using a nucleofector unit (Nucleofector Technology; Amaxa Biosystems, Gaithersburg, MD) according to the manufacturer’s instructions. The optimized protocol used 2µg siRNA and 100 µL of solution T for U251MG cells or R for U118MG and LN229 cells (Amaxa Biosystems) in combination with device program G16 for U251MG cells, T20 for U118MG and X9 for LN229 cells. After transfection, cells were transferred to fresh cell medium supplemented with 5ng/mL fibroblast growth factor and 10 ng/mL epidermal growth factor (Clonetics®, Lonza, Walkersville, MA), in tissue culture dishes or plated in 96-well plates for scratch-wound healing or drug sensitivity assays. Cells in tissue culture dishes were harvested using Trypsin (Gibco, Invitrogen, Burlington, Ontario, Canada) 96 hours after transfection for flow cytometry and western blot analysis.

Stealth RNAi™ sequences against the human EGFR mRNA (Genbank accession #AY588246) and Rictor mRNA (Genbank accession #AY515854), as well as two scrambled Stealth RNAi™ negative control sequences (Low Duplex #1 and Low Duplex #2) with equivalent GC content, were generated by Invitrogen. Stealth RNAi™ sequences are designed by the manufacturer using proprietary algorithms to enhance target specificity and downregulation efficacy. Moreover, chemical modifications ensure that only the antisense strand can participate in the RNAi process, therefore avoiding off-target effects induced by the sense strand. These sequences were also designed to minimize the induction of nonspecific cellular stress response pathways as assessed using in vitro based assays measuring activation of various cytokines. EGFR siRNA sequence (CCU AUG CCU UAG CAG UCU UAU CUA A) and Rictor siRNA sequence (CCU AAU GAA UAU GGC UGC AUC CUU U) were verified using NCBI Basic Local Alignment Search Tool (BLAST) to confirm specificity.

### Scratch-wound Healing and Drug Sensitivity Assay

For the scratch-wound healing assay, cells were grown to 100% confluency in 96-well plates (96 hrs after plating of 1.5−2 ×10^4^ cells depending on the cell line). A scratch was made with a pipet tip and cells were cultured for an additional 24 hrs. Cells were then fixed with 3.5% paraformaldehyde (v/v) in PBS, then stained with eosin (Polyscientific, Bayshore, NY) and photographed on bright field microscope at 5× magnification. Photographs were visualized on Photoshop and scored according to the following criteria: 1: no opening, dense layer of cells and scratch is not visible; 2: no opening but lower cell density along the scratch; 3: scratch ≤0.20 mm; 4: scratch 0.20–0.40 mm; 5: scratch 0.45–0.75 mm; 6: scratch 0.80–1.1 mm; 7: scratch >1.1 mm. The data shown represent the average of a blind scoring from three independent experiments with 8 replicates each. For drug sensitivity assays, cells were plated in 96 well plates (2−5 ×10^3^ cells depending on cell line). Twenty-four hours following cell plating, irinotecan (Sandoz, QC, Canada), vincristine (Novopharm, ON, Canada), and temozolomide (LKT Laboratories inc., St-Paul, MN) were added and 72 hrs after drug addition, MTT reagent (3-(4,5 dimethylthiazol-2-yl)-2,5-diphenyl tetrazolium bromide; Sigma) was added (1.25 mg/mL). The plates were incubated for 3 hrs and the MTT-containing medium was removed and replaced with DMSO. The amount of the blue formazan compound is reflective of the number of living cells and was determined using spectrophotometery (570 nm).

### Immunohistochemistry and Flow Cytometry

Cells were cultured in chamber slides (Nalgene Nunc, Thermo Fisher Scientific, Rochester, NY). Cells were fixed with 3.5% (v/v) paraformaldehyde (Electron Microscopy Sciences, PA) for 15 minutes at −20°C, blocked for 1 hr at 4°C (Odyssey blocking buffer, Rockland, PA) and incubated overnight with rabbit anti-human p(473)-AKT antibody (Cell Signaling Technology, Antibody #9271; rabbit polyclonal; 1∶100 dilution), Rictor (Antibody #NB100–1534 from Novus Biologicals; goat polyclonal; 1∶250 dilution) or EGFR (Antibody #2232 from Cell Signaling Technology; rabbit polyclonal; 1∶50 dilution). Cells were then incubated with Texas Red®-X phalloidin for 30 min at room temperature, goat anti-rabbit (Molecular Probe #A11034, Invitrogen; 1∶200 in blocking buffer) or chicken anti-goat (Molecular Probe #A-21467, Invitrogen; 1∶200 in blocking buffer) Alexa 488 secondary antibodies for 1 hr at room temperature. Nuclei were stained with Draq5 (1∶200 in PBS) or Hoechst 33342 (Sigma, 1∶10000 in PBS) for 30 min at 37°C. Slides were mounted with PBS and imaged for Draq5 (Cy5 filter), Texas Red (TX filter), Hoechst 33342 (UV filter) and Alexa 488 (L5 filter) using a fluorescence microscope (Leica DM6000B, Leica, ON, Canada). A composite color image of these markers was produced (Surveyor software, Objective Imaging Ltd). All fluorescence microscopy images are representative of three independent experiments. For flow cytometry analysis, cells were harvested 96 hr after transfection and fixed overnight in 70% ethanol at −20°C and stained with propidium iodide-containing buffer (50µg/mL; RNase 1 mg/mL; Triton X-100 0.1% in PBS) for an hour on ice and analyzed by flow cytometry.

### Western Blot Analysis

Western immunoblots were obtained from total protein extracts as described previously [34]. The blots were labeled using the following primary antibodies (from Cell Signaling Technology unless otherwise indicated): anti-AKT (Antibody #9272; rabbit polyclonal; 1∶1000 dilution), anti-phosphorylated AKT-Ser473 (Antibody #9271; rabbit polyclonal; 1∶1000 dilution), anti-Rictor (Antibody #2140; rabbit polyclonal; 1∶1000 dilution), anti-EGFR (Antibody #2232; rabbit polyclonal; 1∶1000 dilution), anti-phosphorylated NDRG1 Thr346 (antibody #5482; rabbit monoclonal; 1∶1000 dilution), anti-phosphorylated SGK1 Ser422 (antibody #A0087; rabbit polyclonal; 1∶1000 dilution; Assay Biotech, Sunnyvale, CA), anti-phosphorylated PKCα Ser657 (antibody #06–822; rabbit polyclonal; 1∶4000 dilution; Millipore, Temecula, CA) and anti-β-actin (AC-15; mouse monoclonal; 1∶20 000 dilution; Sigma-Aldrich, Oakville, Ontario, Canada). The secondary antibody used was horseradish peroxidase-conjugated anti-mouse or anti-rabbit IgG (Promega, Madison, WI) diluted 1∶5000. Scanning densitometry (Un-Scan-It software; Silk Scientific, Inc., Orem, UT) was used to quantify band intensities by volume/area integration. Densitometry values were normalized to the corresponding β-actin band, and total AKT in the case of p(473)-AKT. Each western blot figure is representative of three independent experiments and the numbers below each band represent the average of optical densities +/− SEM of all three experiments.

### shRNA Lentivirus Production and U251MG Transduction

Inducible short hairpin RNA constructs (TRIPZ shRNAmir) targeting EGFR (CCCTCCCAGTGCCTGAATACAT, #V2THS_43452), Rictor (CCCAGGCCAGACCTCATGGATA, # V2THS_120392) and a negative control (ATCTCGCTTGGGCGAGAGTAAG, # RHS4743; designed and validated by the manufacturer) were obtained from Open Biosystems (Thermo Fisher Scientific, Rockford, IL). All sequences were verified using NCBI Basic Local Alignment Search Tool (BLAST) to confirm specificity (100% match) or non-specificity (at least 3 or more mismatches against any mammalian gene) of the active and non-active sequences, respectively. RNAintro TRIPZ lentiviral shRNAmir starter kit was used to produce the inducible shRNA. Briefly, and according to the manufacturer’s instructions, one clone from *E. coli* stocks containing TRIPZ shRNAmir plasmids was grown in 5mL 2xLB (low salt) on a shaker at 37°C for 18 hrs. pTRIPZ shRNA DNA was isolated (Qiagen QIAprep Spin Miniprep Kit) and DNA concentration was measured using a NanoDrop® spectrophotometer (ThermoScientific, DE, #ND-001). HEK 293 T cells (5×10^6^ cells) were seeded in a 100 mm plate. Twenty-four hours later, cells were transfected with 37.5µg Trans-lentiviral™ packaging mix, 9µg of pTRIPZ shRNA plasmids and 187.5µL of Arrest-In™ transfection reagent in serum and antibiotic free medium. Medium was replaced with fresh serum and antibiotics-containing medium 4 hrs later. Medium containing viruses was harvested 48 and 72 hrs after transfection, filtered (0.45µm falcon filters, Millex HV; Millipore, Billerica, MA) and concentrated by ultracentrifugation at 23,000 RPM, 4°C for 1.5 hrs.

U251MG cells were plated at 40% density and 24 hrs later, virus stock (multiplicity of infection of 10 for EGFR and Rictor and 20 for negative control) was added to the cells for 4 hrs, then replaced with fresh media and cultured for 48 hrs. Ninety-six hours after transfection, cells were selected for the puromycin resistance gene by adding 50, 15, 22 or 50µg/mL puromycin every three days for negative control, EGFR, Rictor and EGFR/Rictor shRNA-transfected cells, respectively. Puromycin was added for the entire time the cells were maintained in culture. The expression of shRNA was induced in puromycin selected cells by adding 1µg/mL doxycycline (Sigma) in the culture media.

### Orthotopic and Subcutaneous Implantation of shRNA Expressing U251MG Cell Lines

All protocols involving work with live animals were reviewed and approved by the University of British Columbia Animal Care Committee (certificate of approval # A07–0423). Prior to inoculation into animals, the mycoplasma status of all shRNA-transduced cells was confirmed negative by PCR (performed by Idexx Radil Laboratories, Columbia, MO). Negative control shRNA U251MG (U251^Ng2x^), EGFR shRNA U251MG (U251^EGFR^), Rictor shRNA U251MG (U251^Rictor^) and EGFR/Rictor shRNA U251MG (U251^EGFR/Rictor^) cells were implanted (7.5×10^4^ cells) into the brain of Rag2M mice (7–10 weeks old females, 8/Gr). A stereotaxic injection frame (Stoelting Company, Wood Dale, IL) was used to inject cells into the right caudate nucleus-putamen (ML −1.5 mm; AP +1 mm; DV −3.5 mm). Induction of shRNA expression in mice was done 21 days after cell inoculation by dissolving 2 mg/mL doxycycline and 5% sucrose in drinking water. Bottles were made black using a black marker pen as doxycycline is light-sensitive, and water was replaced every three days. Day 21 was chosen as studies previously reported by our laboratory using an mKate2-expressing U251MG line demonstrated the presence of established intracranial tumors at this time point [35]. At the end of the study, animals were imaged using the Maestro fluorescence live animal imaging unit (CRi, Woburn, MA). The animals were anesthetized with isoflurane, and the turbo red fluorescent protein (tRFP) co-expressed with the shRNA sequence in presence of doxycycline was imaged. For the subcutaneous model, cells (5×10^6^) in 4.3 mg/mL Matrigel™ (BD Biosciences, Mississauga, ON, Canada) were implanted subcutaneously (s.c.) into the backs of Rag2M mice (7–10 weeks old females, n = 3). S.c. tumor size was measured 14 days after inoculation by caliper and tumor weights were extrapolated from the measurements (mg = (tumor width^2^ × tumor length)/2).

### Marker Imaging in Orthotopic Tumors and Quantification

Forty-nine days after orthotopic tumor cell inoculation, animals were terminated using CO_2_ asphyxiation and brains were harvested and cryopreserved in OCT on dry ice and stored at −80°C. OCT preserved brains were cryosectioned and 10µm sections were collected from Bregma +1.0 location. Sections were fixed with 3.5% paraformaldehyde (Electron Microscopy Sciences, PA) for 15 minutes at room temperature, then blocked with blocking buffer (Odyssey blocking buffer, Rockland, PA) for 1 hour at room temperature, and stained with p(473)AKT (Antibody #4060 from Cell Signaling Technology; rabbit monoclonal; 1∶50 dilution), Rictor (Antibody #NB100–1534 from Novus Biologicals; goat polyclonal; 1∶250 dilution), EGFR (Antibody #2232 from Cell Signaling Technology; rabbit polyclonal; 1∶50 dilution), Ki67 (Invitrogen #18–0191z; 1∶100) and Alexa 488 goat anti-rabbit (Molecular Probe #A-11034, Invitrogen; 1∶200 in blocking buffer) or Alexa 488 chicken anti-goat (Molecular Probe #A-21467, Invitrogen; 1∶200 in blocking buffer) secondary antibodies. Primary antibodies were incubated on sections overnight at 4°C, and secondary antibodies for 1 hr at room temperature. Nuclei were stained with Hoechst 33342 (Sigma; 5µg/mL) for 30 min at 37°C. Slides were washed with PBS and imaged for Alexa 488 (L5 filter), tRFP (CY3 filter) and Hoechst 33342 (UV filter) using a robotic fluorescence microscope (Leica DM6000B, Leica, ON, Canada). Acquired images were quantified using an in-house segmentation algorithm (MATLAB, The Mathworks, Natick, MA) and a composite color image of these markers was produced (Surveyor software, Objective Imaging Ltd.). EGFR, Rictor, p(473)-AKT and Ki67 markers are expressed as the number of positive pixels in the tumor area divided by the number of Hoechst (nuclei stain) positive pixels.

### Statistical Analysis

All statistical data were collected using GraphPad Prism (San Diego, CA). Parametric analysis was done using standard deviation, mean and n, in a multivariate one-way analysis (ANOVA) with Tukey’s post-tests. All data are shown as mean ± S.E.M.

## Results

### The Combined Silencing of EGFR and Rictor Results in a Reduction in Cell Migration and an Increase in Cell Sensitivity to Chemotherapeutic Drugs

**Figure 1:**
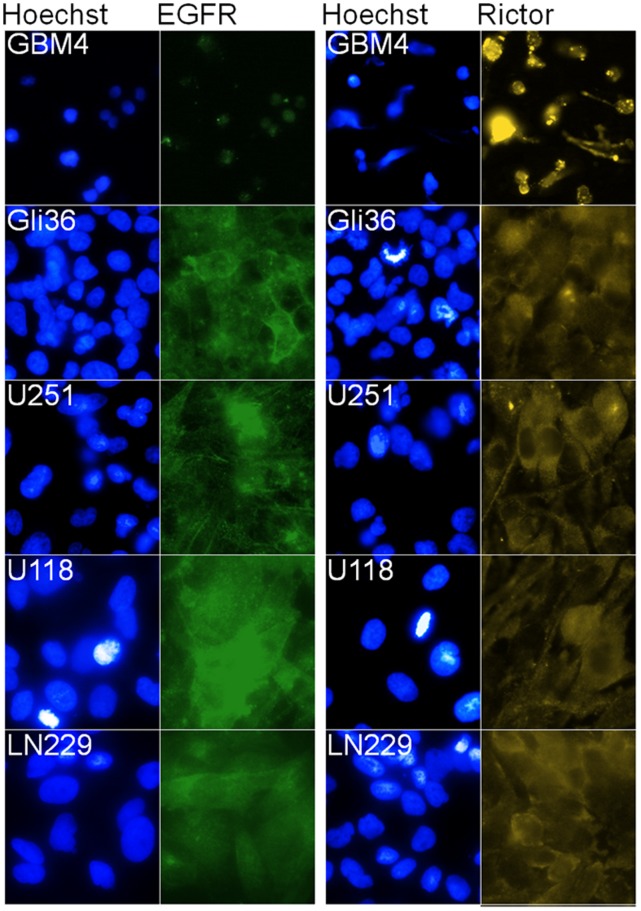
Fluorescence micrographs showing EGFR (Alexa 488; green), Rictor (Alexa 488; yellow) and cell nuclei (Hoechst 33342; blue) in GBM4 GBM-derived cancer stem-like cell line, and Gli36, U251MG, U118MG and LN229 GBM cell lines.

**Figure 2:**
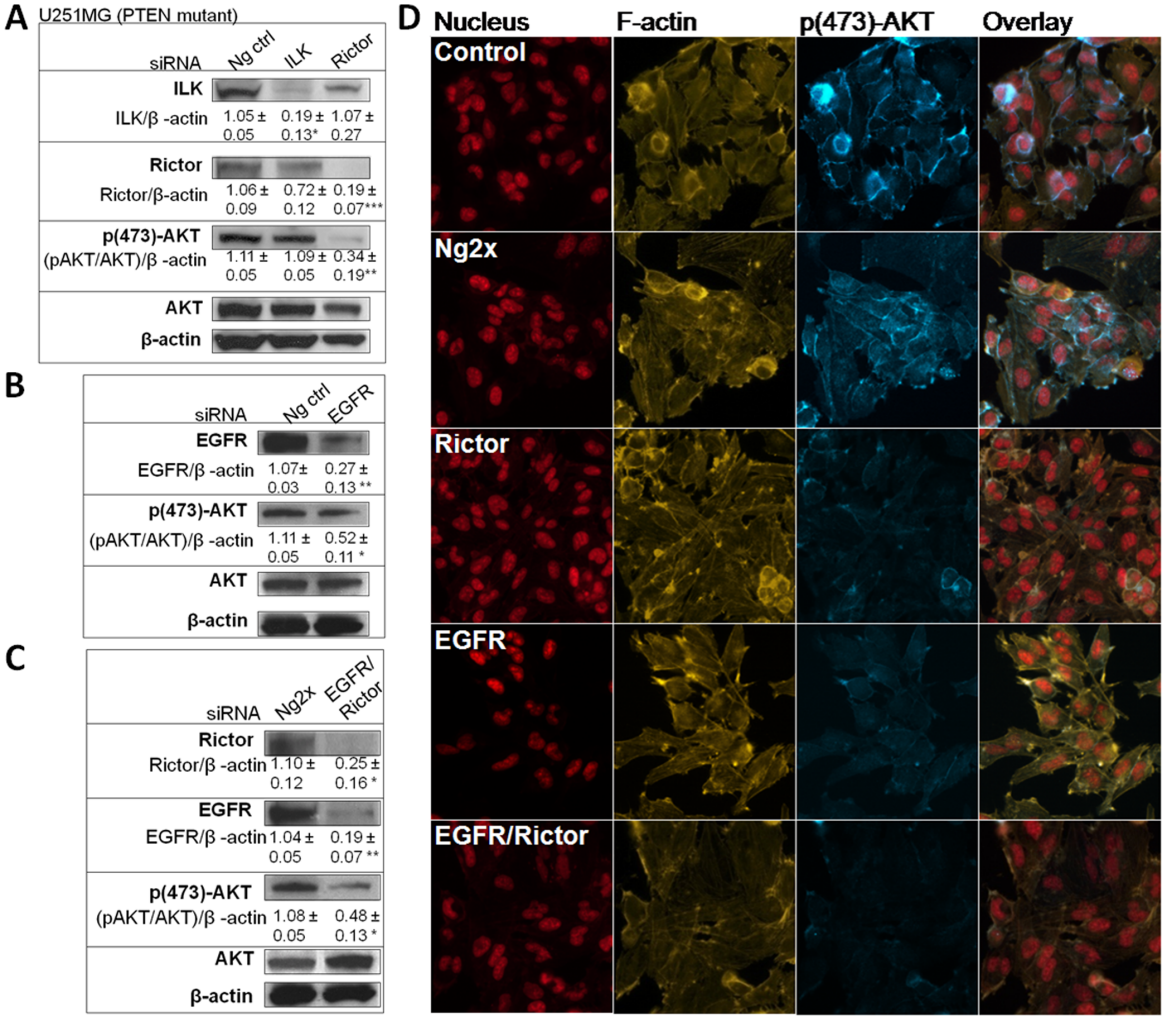
Transfection of siRNA sequences specific to Rictor and EGFR results in downregulation of their respective proteins in U251MG cell line. Optical density values shown are expressed relative to values obtained from untreated cells, which correspond to a value of 1. **a**) Representative immunoblots showing ILK, Rictor, p(473)AKT, AKT and β-actin from U251MG cells 96hrs after transfection of siRNA against ILK or Rictor or the negative control sequence (Ng ctrl). **b**) Representative immunoblots showing EGFR, p(473)AKT, AKT and β-actin from U251MG cells 96hrs after transfection of siRNA against EGFR or the negative control sequence (Neg ctrl). **c**) Representative immunoblots showing Rictor, EGFR, p(473)AKT, AKT and β-actin from U251MG cells 96 hrs after transfection of the combination of Rictor and EGFR siRNAs or the combination of two negative control sequences (Ng2x). Optical density values shown under each band represent the average obtained from three independent experiments (±SEM) normalized to β-actin, and AKT in the case of p(473)AKT. **d**) Representative fluorescence photomicrograph (n = 3) of U251MG cells showing nuclei (Draq5; red), F-actin (Texas red phalloidin; Yellow), and p(473)-AKT (Alexa 488; blue) 96 hrs after transfection of siRNA against Rictor, EGFR, the combination of Rictor and EGFR, or the combination of two negative sequences (Ng2x).

High levels of Rictor expression were found in a panel of four GBM cell lines (Gli36, U251MG, LN229 and U118MG) and one GBM-derived cancer stem-like cell line (GBM4 [36]) (Fig. 1). Rictor is involved in the phosphorylation of kinases that are particularly relevant in the context of malignancy: PKCα (serine 657 site) and AKT (serine 473 site; p(473-AKT)) [17], [18]. Rictor and its partner mTOR were suggested to be necessary for the phosphorylation of p(473)-AKT [17]. Therefore, p(473)-AKT was selected as a key marker for the functional signaling consequences associated with Rictor silencing. However, research from our laboratory and colleagues have suggested that other proteins such as Integrin-Linked Kinase (ILK) can also participate in AKT phosphorylation [37]–[39]. Thus, the level of p(473)-AKT in U251MG cells following siRNA-mediated silencing of Rictor was evaluated and compared with p(473)-AKT levels following silencing of ILK. Transfection of the negative control sequence had no significant impact on the level of proteins that were measured. The representative immunoblots provided in Figure 2 also include the averaged optical density assessments determined from 3 separate experiments (numbers under each band). The values indicated are expressed relative to those obtained from untreated cells (standardized to a value of 1). These data indicate that the selected siRNA sequences designed to silence Rictor or ILK were effective at silencing Rictor and ILK, respectively. Following transfection of siRNA specific to ILK, the protein level of ILK was decreased by 81% (p<0.05). The transfection of the siRNA sequence specific to Rictor reduced Rictor levels by 81% (p<0.001). Under the conditions used here, no reduction in p(473)-AKT were noted when ILK expression was silenced in these cells. However, Rictor silencing resulted in a 66% (p<0.01) reduction in p(473)-AKT expression.

High levels of EGFR expression were also found in all four GBM cell lines tested (Gli36, U251MG, U118MG and LN229), consistently with previously published observation [40]–[42]. In contrast, low levels of EGFR expression were seen in GBM4 GBM-derived cancer stem-like cell line (Fig. 1), and this observation is also consistent with microarray-based comparative genomic hybridization data obtained from colleagues [43]. Silencing of EGFR in U251MG cells (Fig. 2B) could be achieved following transfection of EGFR siRNA (73% reduction in protein levels, p<0.01, compared to control cells) and this was associated with a 48% reduction in p(473)-AKT levels (p<0.05). As noted in Fig. 2C, when the U251MG cells were transfected with Rictor siRNA and EGFR siRNA, the levels of EGFR and Rictor downregulation were comparable to those observed in cells transfected with the individual siRNA sequences (81% and 75%, respectively; p<0.05). The combination of Rictor and EGFR silencing was associated with a 52% (p<0.05) decrease in p(473)-AKT levels that was comparable to that of cells transfected with the individual siRNAs. Representative fluorescence photomicrographs showing the p(473)AKT staining in cells following Rictor and/or EGFR siRNAs transfection have been provided Fig. 2D. These data suggest that significant reductions in p(473)-AKT are achievable when Rictor and EGFR are silenced either alone or in combination. F-actin staining was used to reveal the structure of the cytoplasm in these photomicrographs.

**Figure 3:**
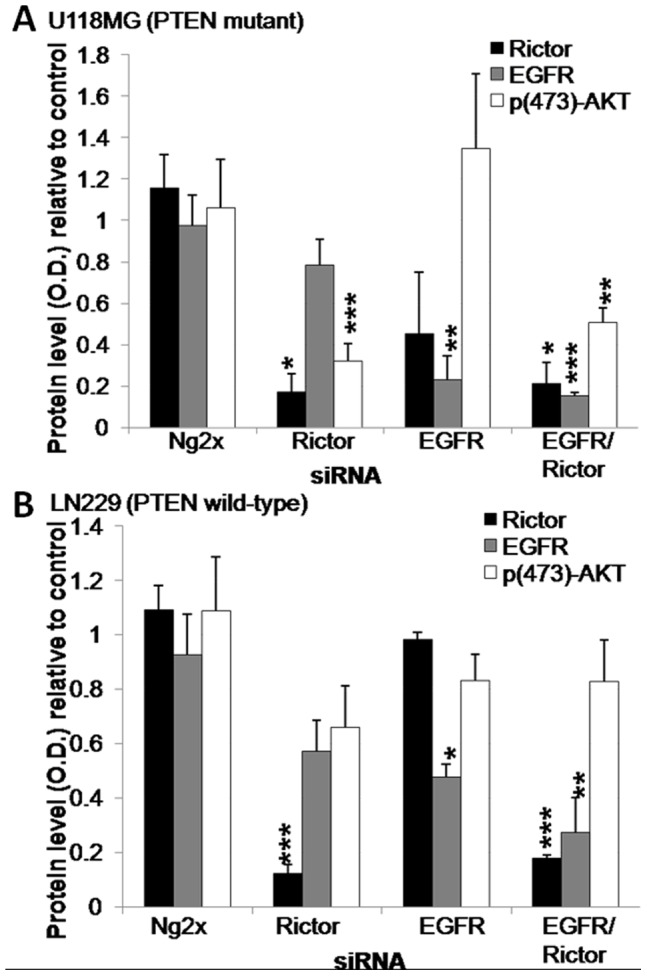
Transfection of siRNA sequences specific to Rictor and EGFR results in downregulation of their respective proteins in U118MG and LN229 GBM lines. Histograms showing Rictor, p(473)AKT, AKT and β-actin protein levels relative to untreated cells. Optical density values were normalized to the β-actin value, and the AKT value in the case of p(473)AKT, and represent the average obtained from three independent experiments from **a**) U118MG and **b**) LN229 cells 96 hrs after transfection of siRNA against Rictor, EGFR, the combination of Rictor and EGFR siRNAs or the combination of two negative control sequences (Ng2x). *p-value ≤0.05; **p-value ≤0.01; ***p-value ≤0.001.

The experiments summarized above for U251MG cells were also completed in U118MG and LN229 cells. The LN229 cell line expresses wild-type PTEN, while U251MG and U118MG carry mutant forms of PTEN (caused by inactivating mutations such as a frame shift at codon 241 and an exon/intron 8 splicing defect, respectively [44]). As summarized in Figure 3, Rictor siRNA transfection resulted in an 83% (p<0.05) and 88% (p<0.001) reduction in Rictor protein levels in U118MG and LN229 lines, respectively. The levels of protein suppression seen in both cell lines were similar to what was obtained with the U251MG cell line. Interestingly, Rictor silencing was associated with significant reductions in p(473)-AKT levels only in the PTEN mutant cell lines (U251MG and U118MG cells). Indeed, in U118MG cells, silencing of Rictor resulted in a 68% reduction of p(473)-AKT levels (p<0.001; Fig. 3a), comparably to the 67% reduction in p(473)-AKT levels noted in the U251MG cells. In LN229 cells, a mean suppression of 25% was noted for p(473)AKT but this decrease was not statistically significant when compared to the controls (Fig. 3B). Transfection of EGFR siRNAs resulted in a 77% (p<0.01) and 52% (p<0.05) reduction in EGFR protein levels in U118MG and LN229 lines, respectively. No change in p(473)AKT was observed in U118MG and LN229 lines following EGFR siRNA transfection (Fig. 3A–B). When U118MG and LN229 cells were transfected with siRNAs targeting Rictor and EGFR, downregulation of EGFR and Rictor was comparable to that achieved when the siRNA sequences were used alone (Fig. 3). Once again, significant p(473)-AKT suppression was only observed in the PTEN mutant U118MG cell line (49%; p<0.01) and this effect is likely to be a consequence of Rictor silencing.

The results described above provided the basis for studies evaluating how Rictor and/or EGFR silencing influence GBM cell migration *in vitro* and exploring whether silencing of these targets influenced cell sensitivity to conventional agents known to exert activity in patients with GBM. It is important to note, however, that EGFR and Rictor silencing, alone or in combination, had little effect on cell viability and proliferation over the course of 96 hrs *in vitro*. These data (not shown) were obtained using the MTT assay described in the Materials and Methods. In U251MG and LN229 cells lines transfected with both Rictor and EGFR siRNA, there was a small reduction (<14%) in the number of viable cells measured after a 96hr incubation when compared to negative controls. The combination had no measurable impact on U118MG cell viability (data not shown). Further, flow cytometric analysis of the sub-G1/G0 fraction at the end of the 96 hr time course indicated that the siRNA transfections (Ng control, Ng2x control, Rictor, EGFR or Rictor/EGFR) did not increase the apoptotic or dead cell fraction when compared to untreated controls (data not shown).

**Figure 4:**
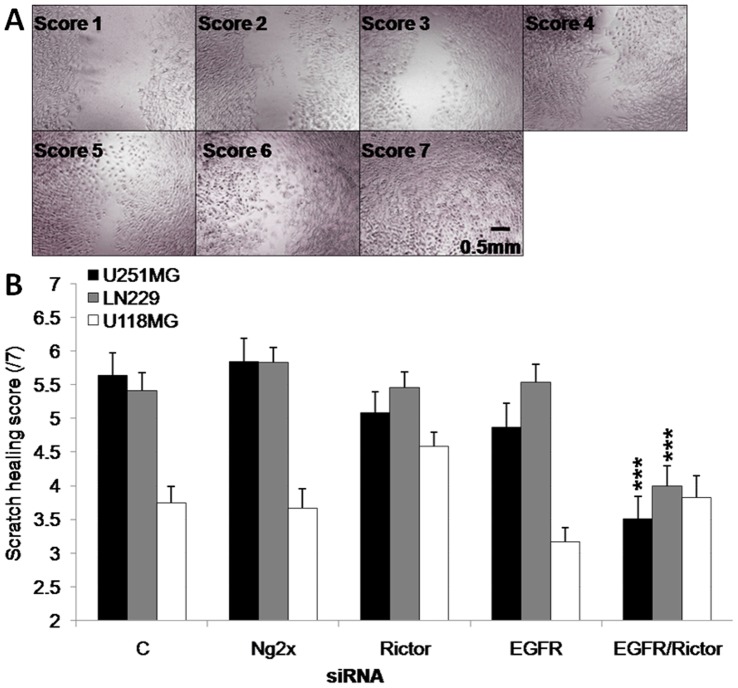
The combination of EGFR and Rictor silencing results in a reduction in cell migration. **a**) Example of scoring chart for the scratch-wound healing assay. **b**) Scratch width scoring of U251MG, U118MG and LN229 cells 96 hrs after transfection of siRNA against Rictor, EGFR, the combination of Rictor and EGFR or the combination of two negative control sequences (Ng2x), obtained from three independent experiments. ***p-value ≤0.001 compared to untreated cells.

One important feature of GBM cells is the capacity of these cells to migrate into brain tissue. Cell migration occurs through highly coordinated processes including cell motility, which involves reorganization of the actin cytoskeleton [45]. It has already been demonstrated that activation of EGFR promotes cell motility through several processes including modulation of the RHO GTPases and PLC-γ [45]–[47]. Moreover, Rictor was shown to be involved in reorganization of the actin cytoskeleton through its downstream effector PKC-α [48]–[52]. For these reasons, it was anticipated that Rictor and/or EGFR silencing might inhibit cell motility. A scratch-wound healing assay was used (as described in the Materials and Methods) to study directional cell motility. Changes in the wound width were determined in a blinded fashion 24 hrs after wounding according to criteria described in Methods and illustrated in Figure 4A. It is important to note that data from this assay can be difficult to interpret if the cell populations being studied exhibit different proliferation rates. As noted above, the treated cell populations exhibited similar proliferation rates over 96 hrs, so changes in scoring should be reflective of changes in cell migration rates; higher scores (up to 7) represent cells with greater migration capability when compared to cells with lower scores, which exhibit reduced migration capability (Fig. 4A).

Scratch-wound healing data were collected for three cell lines (U251MG, U118MG and LN229) and these data have been summarized in Figure 4B. When considering the control cells, it is clear that U118MG cell migration was much lower than that observed in untreated U251MG and LN229 cell lines. Transfection with two negative control sequences (Ng2x) had no effect on cell migration in the three lines when compared to control cells. When the cell lines were transfected with siRNAs targeting Rictor or EGFR alone, no significant changes were observed in wound healing capability (Fig. 4B). However, for the cell lines which exhibited the greatest migration capability (U251MG and LN229), silencing of Rictor and EGFR together resulted in significant (p<0.001) reductions in migration. More specifically, control cell populations exhibited a mean score on the scratch assay of 5.4 to 5.7, while cell populations where both Rictor and EGFR were silenced exhibited scores of 3.5 to 4.0 (Fig. 4B).

**Figure 5:**
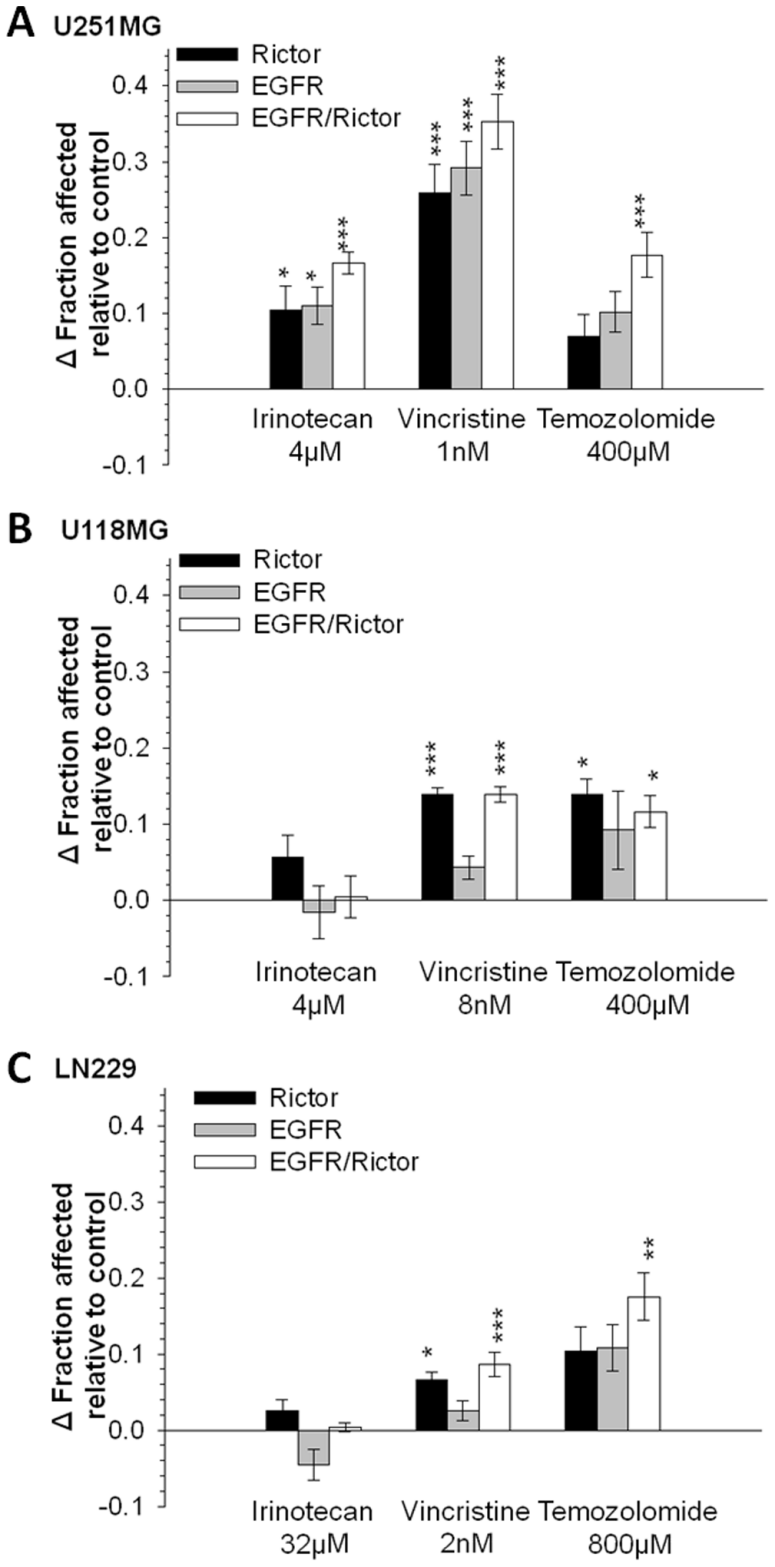
The combination of EGFR and Rictor silencing results in an increase in cell sensitivity to chemotherapeutic drugs. Changes in fraction affected measured by MTT assay of **a**) U251MG, **b**) U118MG and **c**) LN229 cells 96 hrs after transfection of siRNA against Rictor, EGFR or the combination of Rictor and EGFR, and treated with irinotecan, vincristine or temozolomide at EC50 concentrations determined for control non-transfected cells at 72 hrs. Transfection of the two negative control sequences did induce an increase in Fa of LN229 exposed to vincristine of 0.13 compared to non-transfected cells and in this case only, changes in Fa are expressed relative to Ng2x-transfected cells. For all other cases, changes in Fa are expressed relative to control non-transfected cells. *p-value ≤0.05; **p-value ≤0.01; ***p-value ≤0.001.

Two of the basic tenets behind this research are: i) target silencing alone will not be sufficient to achieve optimal therapeutic results and ii) targeted therapeutics, such as siRNA therapeutics, will be used in combination with existing treatments which are known to provide measurable therapeutic benefits to patients. In consideration of these tenets, it is important to establish that target silencing does not reduce the sensitivity of tumor cells to conventional treatment modalities such as chemotherapeutic drugs. It is also valuable to assess whether target silencing can increase the sensitivity of tumor cells to these chemotherapeutic agents. Thus, GBM tumor cell lines transfected with EGFR siRNA, Rictor siRNA, or the combination of both, were exposed *in vitro* to chemotherapeutic drugs commonly used in for treatment of patients with GBM including: irinotecan [53], [54], vincristine [55] and temozolomide [56]. Complete dose- response curves for each drug were generated in control cells (i.e. not transfected) using a 72 hour time course (data not shown). These data were used to determine the drugs EC_50_ i.e. the concentration of drug where cell viability/proliferation was inhibited by 50% when compared to untreated cells (fraction affected or Fa of 0.5). Subsequently, cells transfected with siRNA targeting Rictor and EGFR alone or in combination were exposed (24 hrs after cell transfection) to the drugs at their approximate EC_50_ (see Figure 5) and cell viability was determined 72 hrs later. The results, summarized in Figure 5, are shown as a relative increase or decrease (Δ) in Fa when compared to control cells (non-transfected or Ng2x-transfected cells) that were treated with the same dose. The data suggest that the combination of Rictor and EGFR silencing results in increased drug sensitivity in all examples, with the exception of LN229 and U118MG exposed to irinotecan. In these cases, drug sensitivity in the siRNA transfected cells was not significantly different from that observed in control cell populations. Significant increases in Fa are exemplified by the results obtained with the U251MG cells exposed to 1 nM Vincristine (Fig. 5A). Silencing of EGFR or Rictor increased the Fa following vincristine exposure by 0.26 and 0.29 (p<0.001), respectively, compared to the non-transfected control cell population. When both EGFR and Rictor were silenced in combination, the increase in Fa following vincristine exposure was 0.35 (p<0.001), suggesting that the dose of vincristine that would cause a 50% decrease in cell viability and proliferation of non-transfected cells produced a 85% decrease under conditions where EGFR and Rictor were silenced simultaneously.

### Combined Silencing of Rictor and EGFR in an Orthotopic Model of GBM Leads to Tumor Regression

In aggregate the *in vitro* data summarized above suggested that the combined silencing of EGFR and Rictor decreased cell migration in U251MG and LN229 cells and that dual silencing of these targets did not reduce sensitivity (and in many cases actually increased sensitivity) to selected chemotherapeutic agents. These data were sufficient to justify further studies using an *in vivo* model of GBM. The therapeutic potential of the combined silencing of EGFR and Rictor was assessed *in vivo* in an orthotopic model of GBM generated by intracranial inoculation of shRNA-expressing U251MG cells. U251MG cells were transduced using a lentiviral system designed to express shRNA sequences specific to EGFR (U251^EGFR^), Rictor (U251^Rictor^) or both (U251^EGFR/Rictor^). Control cells were produced by transduction with the negative control sequences (U251^Ng2x^) as described in the Methods. The expression system used was inducible in the presence of doxycycline. This facilitated characterization of the transduced cell lines prior to and following induction of shRNA expression. The lentiviral system used was also designed to co-express Turbo Red Fluorescent Protein (tRFP) upon doxycycline induction. tRFP expression was assessed using non-invasive imaging methods or by fluorescent microscopy of thin sections, as a means of confirming shRNA expression.

**Figure 6:**
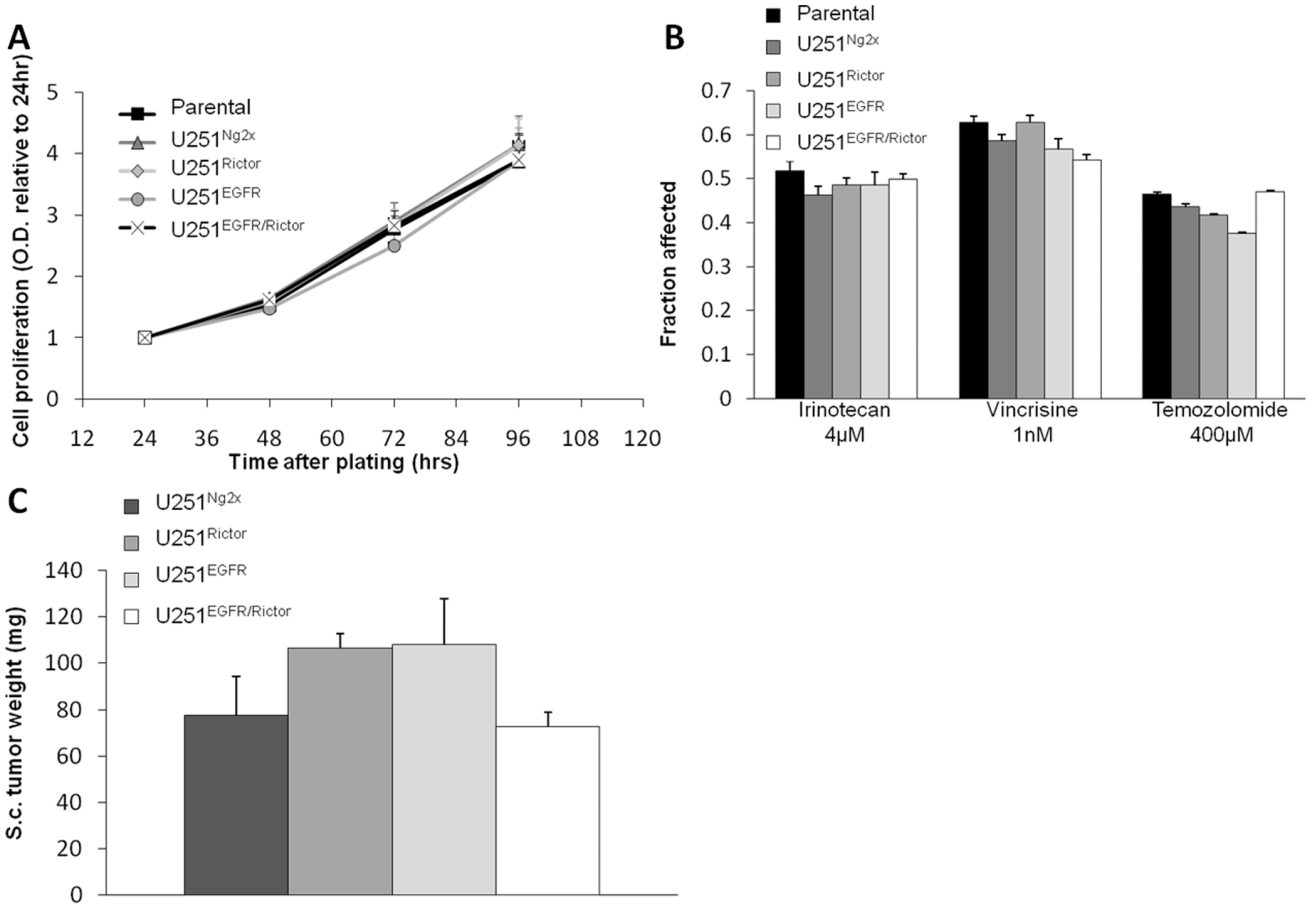
Un-induced lentiviral shRNA-transduced cell lines behave similarly to each other. **a**) Relative cell proliferation measured by MTT assay (24–96 hr time points) of U251^Ng2x^, U251^Rictor^, U251^EGFR^ and U251^EGFR/Rictor^ in the absence of doxycycline. Optical density values are normalized to values obtained at 24 hrs. **b**) Fraction affected (normalized to untreated cells) measured by MTT assay of U251^Ng2x^, U251^Rictor^, U251^EGFR^ and U251^EGFR/Rictor^ in absence of doxycycline and treated with irinotecan, vincristine and temozolomide for 72 hrs. For *in vitro* data, all values shown represent the average from three independent experiments. **c**) Subcutaneous tumor weight 14 days after inoculation of U251^Ng2x^, U251^Rictor^, U251^EGFR^ and U251^EGFR/Rictor^ in the absence of doxycycline (3 mice/group).

It was important to determine that lentivirus transduction did not influence the behavior of the transduced U251MG cells prior to addition of doxycyline. Growth curves of the four selected cell lines (U251^Rictor^, U251^EGFR^, U251^EGFR/Rictor^, or U251^Ng2x^ cells) in the absence of doxycycline are provided in Figure 6A, where increases in cell number were followed over a 96hr time course. The results demonstrate that all four cell lines have identical growth rates *in vitro* when compared to the parental cell line. Further, the sensitivity of these cell lines to irinotecan, vincristine and temozolomide was also assessed in the absence of doxycycline. The results, summarized in Fig. 6B, indicate that the fraction of affected cells following addition of the indicated drugs was not significantly different in the transduced cells when compared to the parental cells (filled bar), regardless of the drug concentration used. Subcutaneous inoculation of all four cell lines in Rag2M mice also confirmed that U251^Rictor^, U251^EGFR^, U251^EGFR/Rictor^, or U251^Ng2x^ exhibited comparable *in vivo* growth rate in the absence of doxycycline (Figure 6C). It should be noted that previous studies by our laboratory have demonstrated that s.c. inoculation of U251 parental line results in tumors of sizes comparable to that of all four transduced lines for the time point shown (14 days) (data not shown). However, in order to reduce the number of animals used, it was decided that for all in vivo experiments included in this study, the U251Ng2x line would be used as the control in the *in vivo* studies.

**Figure 7:**
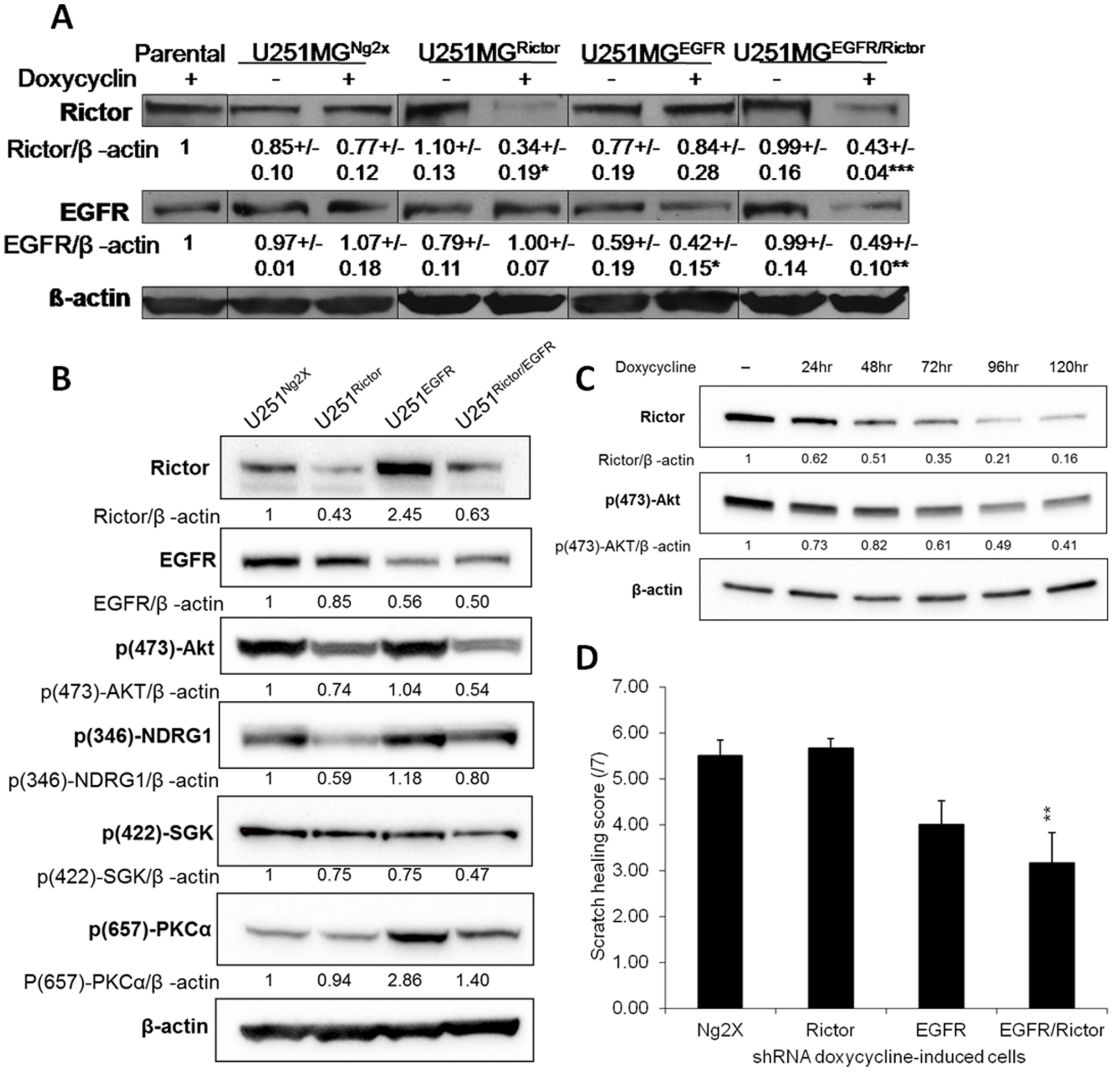
Induction of lentiviral shRNA-transduced cells results in downregulation of corresponding proteins *in vitro* and downstream effectors, and reduction in cell migration. **a**) Representative immunoblots showing Rictor, EGFR and β-actin from parental U251MG cells, U251^Ng2x^, U251^Rictor^, U251^EGFR^ and U251^EGFR/Rictor^ in the absence (-) or presence (+) of doxycycline. Average of band optical density normalized to β-actin from three independent experiments (+/−SEM), and expressed as relative to values obtained from parental cells, is shown under each band. *p-value ≤0.05; **p-value ≤0.01; ***p-value ≤0.001 compared to parental cells. **b**) Representative immunoblots showing Rictor, EGFR, p(473)-AKT, p(346)-NDRG1, p(422)-SGK, p(657)-PKCα and β-actin from U251^Ng2x^, U251^Rictor^, U251^EGFR^ and U251^EGFR/Rictor^ exposed to doxycycline for 72hrs. Average of band optical density normalized to β-actin and expressed as relative to values obtained from U251^Ng2x^ is shown under each band. **c**) Representative immunoblots showing Rictor, p(473)-AKT and β-actin from U251^Rictor^ in the absence (-) of doxycycline or exposed to doxycycline for 24–120 hrs. Average of band optical density normalized to β-actin and expressed as relative to values obtained from U251^Ng2x^ in the absence of doxycycline is shown under each band. **d**) Scratch width scoring of U251^Ng2x^, U251^Rictor^, U251^EGFR^ and U251^EGFR/Rictor^ 18hrs after scratching in presence of doxycycline and after pre-incubation with doxycycline for 72 hrs.**p-value ≤0.01 compared to U251^Ng2x^ cells.

To confirm that shRNA expression caused reductions in EGFR and/or Rictor protein levels following addition of doxycyline, western immunoblot analysis was completed and these data have been summarized in the representative immunoblot provided in Figure 7. The results shown in Figure 7A (averaged optical density under each band is provided relative to values obtained from untreated controls for 3 independent experiments) show EGFR and Rictor expression in the parental U251MG line as well as U251^Rictor^, U251^EGFR^, U251^EGFR/Rictor^, and U251^Ng2x^ cells in the presence (+) or absence (-) of doxycycline. The protein levels of Rictor and EGFR in the U251^Ng2x^ cells, in the presence or absence of doxycycline, were not significantly different from the parental cells. In the absence of doxycycline, the levels of Rictor in the U251^Rictor^ and U251^EGFR/Rictor^ cells were not significantly different from that observed in the parental or the U251^Ng2x^ lines. When U251^Rictor^ and U251^EGFR/Rictor^ cell lines were exposed to doxycycline, the levels of Rictor were reduced by 66% and 57% for U251^Rictor^ and U251^EGFR/Rictor^, respectively (p<0.05). In the absence of doxycycline, a slight reduction in EGFR levels was observed compared to the parental or the U251^Ng2x^ lines, although this difference was not significant. In U251^EGFR/Rictor^ cells in absence of doxycycline, EGFR levels were also not significantly different from that observed in the parental or the U251^Ng2x^ lines. When the U251^EGFR^ and U251^EGFR/Rictor^ cells were cultured in the presence of doxycycline, the levels of EGFR were significantly (p<0.05) reduced (58% and 51%, respectively). Expression of tRFP following doxycycline induction was also confirmed (by fluorescence microscopy) in the U251^Rictor^, U251^EGFR^, U251^EGFR/Rictor^, or U251^Ng2x^ cell lines (data not shown), providing an additional demonstration of the functionality of the inducible system *in vitro*.

In order to confirm the specificity of the constructs used, the effects of doxycycline-induced Rictor shRNA were assessed on a panel of four effectors reported to be regulated by Rictor: p(473)-AKT [17], [18], phosphorylated NDRG1 at the threonine 346 site (p(346)-NDRG1) [57] phosphorylated SGK at the serine 422 site (p(422)-SGK) [57], and phosphorylated PKCα at the serine 657 site (p(657)-PKCα) [18] (Figure 7B). Expression of Rictor shRNA reduced the level of p(473)-AKT, p(346)-NDRG1 and p(422)-SGK, but did not impact the level of p(657)-PKCα. Combined expression of EGFR and Rictor shRNA further reduced the level of p(473)-AKT and p(422)-SGK. The level of suppression of p(346)-NDRG1 was reduced in U251^EGFR/Rictor^ cells compared to U251^Rictor^, but increases in NDRG1 were previously reported to be associated with EGFR suppression [58]. Moreover, the impact of expression of Rictor shRNA on Rictor expression and p(473)-AKT levels was shown to be dependent on doxycycline exposure time (Figure 7C). Interestingly, however, p(657)-PKCα level was increased in U251^EGFR^ and U251^EGFR/Rictor^ cells compared to U251^Ng2x^ and U251^Rictor^ cells, which could possibly be explained by EGFR suppression-induced compensatory mechanisms in these cells. Taken together, these data confirm that the doxycycline-inducible shRNA construct against Rictor is specific to its target and induces expected effects on most of Rictor downstream effectors.

Proliferation and viability of doxycycline-induced cells was assessed over a 96 hrs period and, similarly to what was obtained with the transfection of siRNA against the same targets, proliferation and viability of these cells was not affected significantly (data not shown). Scratch-wound healing data were also collected for U251^Ng2x^, U251^Rictor^, U251^EGFR^ and U251^EGFR/Rictor^ cells after exposure to doxycycline for 96 hours using the same scoring system described in Figure 4. These data are shown in Figure 7D. Silencing of Rictor and EGFR together using the shRNA inducible system resulted in significant (p<0.01) reductions in migration. More specifically, U251^Ng2x^ cells exhibited a mean score on the scratch assay of 5.5, while U251^EGFR/Rictor^ cells exhibited a mean score of 3.17 (Fig. 7D). Doxycyline-induced U251^Rictor^ and U251^EGFR^ cells did not exhibit significant reductions in their migration capacity. These data support what was observed with the transfection of siRNA *in vitro*, and strongly suggest that combined silencing of EGFR and Rictor reduces cell migration capacity.

**Figure 8:**
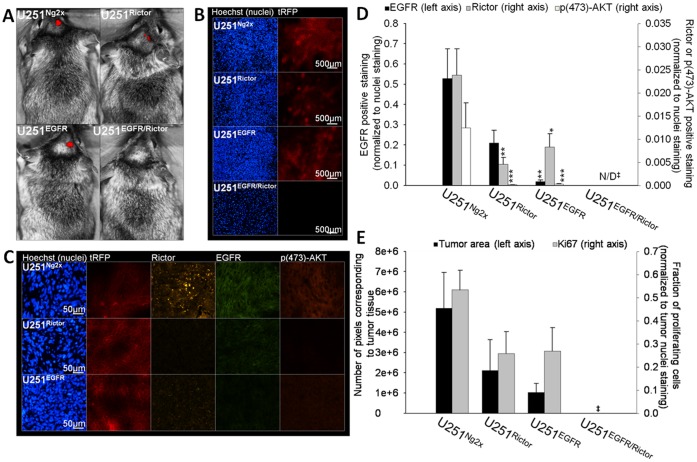
The combined silencing of Rictor and EGFR *in vivo* results in a complete inhibition of tumor growth. U251^Ng2x^, U251^Rictor^, U251^EGFR^ and U251^EGFR/Rictor^ cells were implanted into the right caudate nucleus-putamen of Rag2M mice (n = 6−8). Induction of shRNA expression in mice was initiated on day 21 by dissolving 2 mg/mL doxycyline and 5% sucrose in drinking water. **a**) On day 49, animals were imaged by Maestro™ fluorescence imaging unit for the expression of tRFP co-expressed with the shRNA sequences upon doxycycline-induced expression. Mice were then terminated and brains were harvested, sectioned and stained for nuclei, Rictor, EGFR and p(473)-AKT and imaged for all markers in addition to tRFP by robotic fluorescence microscopy. No tumor was detected in the U251^EGFR/Rictor^ group. **b**) A representative brain section from U251^Ng2x^, U251^Rictor^, U251^EGFR^ and U251^EGFR/Rictor^ tumor groups is shown: tRFP (red) and Hoechst (blue). **c**) A representative tumor section from U251^Ng2x^, U251^Rictor^ and U251^EGFR^ tumor groups is shown: nuclei (blue), rRFP (red), Rictor (yellow), EGFR (green) and p(473)-AKT (orange). **d**) The expression of EGFR (left axis), Rictor (right axis) and p(473)-AKT (right axis) in U251^Ng2x^, U251^Rictor^, U251^EGFR^ tumor sections were quantified (positive staining normalized to Hoechst nuclei staining). **e**) Tumor sizes were estimated by quantification of tumor areas in brain sections from all groups (left axis). The expression of the proliferation marker Ki67 in the tumor (proliferating fraction) was also quantified (right axis). *p-value ≤0.05; **p-value ≤0.01; ***p-value ≤0.001 compared to control untreated cells. ‡: No tumor was detected in the U251^EGFR/Rictor^ group.

The effects of EGFR and Rictor downregulation alone or in combination were assessed in GBM models created following orthotopic inoculation of U251^Rictor^, U251^EGFR^, U251^EGFR/Rictor^, or U251MG^Ng2x^ cells. Twenty-one days after cell inoculation, mice were given doxycycline *ad libitum* in drinking water as described in the Methods. Four weeks after doxycycline dosing was initiated (49 days after tumor cell inoculation), mice were imaged (Maestro™ fluorescence imager) to determine whether tRFP was expressed. Representative images, provided in Fig. 8A, show that the expression of tRFP could be detected in animals inoculated with U251^Rictor^, U251^EGFR^, and U251^Ng2x^ cells. However, no tRFP was detected in animals inoculated with U251^EGFR/Rictor^ cells. All mice were terminated after imaging and their brains were harvested and sectioned as described in the Materials and Methods. Figure 8B provides representative images of brain sections isolated from each group. In animals inoculated with U251^Rictor^, U251MG^EGFR^, and U251^Ng2x^ cells, tumors were detected and can be seen in Figure 8B by the presence of dense Hoechst-stained nuclei (blue) and tRFP (red) expression. No tumors could be detected in all eight animals inoculated with the U251^EGFR/Rictor^ cells.

Figure 8C provides representative images of tumor sections isolated from U251^Rictor^, U251^EGFR^, and U251^Ng2x^ tumor-bearing animals. These sections were imaged for tRFP (red), EGFR (green), Rictor (yellow) and p(473)-AKT (orange). tRFP expression was confirmed in all three tumor groups. A decrease of 96% (p<0.001) in EGFR staining (normalized to Hoechst nuclei staining) was measured in tumors from animals inoculated with U251^EGFR^ cells (Fig. 8C and D). Rictor staining (normalized to Hoechst nuclei staining) was decreased by 81% (p<0.01) in tumors from animals inoculated with U251^Rictor^ cells. A 65% reduction (p<0.05) in Rictor staining was also observed in tumors from animals inoculated with U251^EGFR^ cells, which was not unexpected as Rictor is located downstream of EGFR [25]. Finally, a 97–99% reduction (p<0.05) in p(473)-AKT staining (normalized to Hoechst nuclei staining) was measured in tumors from animals inoculated with U251^Rictor^ or U251^EGFR^ cells. The fraction of proliferating tumor cells was also determined in tumors from animals inoculated with U251^Rictor^, U251^EGFR^, and U251^Ng2x^ cells by quantification of Ki67 positive staining (normalized to nuclei staining). These data, summarized in Fig. 8E, indicate that there were no significant changes in the fraction of proliferating cells when the three groups were compared. Tumor size was estimated on the basis of histology data and these data (estimations of pixel number per tumor area of each section) have also been summarized in Fig. 8E. While the tumors in animals inoculated with U251^Rictor^ and U251^EGFR^ cells were smaller than those from animals inoculated with U251^Ng2x^ cells, these differences were not significant. As noted already, following doxycyline induction, no tumors were detected in the animals inoculated with the U251^EGFR/Rictor^ cells, an effect confirmed by two additional independent sets (n = 4−8) of intracranial inoculation with this cell line.

## Discussion

The importance of mTOR and EGFR signaling in GBM has been reported and discussed extensively. Despite strong preclinical rationale indicating that targeting of EGFR and mTOR should provide therapeutic benefit, these studies have resulted in only modest benefits. More specifically, mTOR small molecule inhibitors [59], [60] have shown no evidence of therapeutic activity in recurrent GBM disease (reviewed in [19]). Use of the EGFR tyrosine kinase inhibitor gefitinib showed no measurable responses [61], while treatment with the EGFR inhibitor erlotinib resulted in only a 6–11% objective response rate (partial or complete response), albeit these were not linked to EGFR overexpression [62], [63]. Perhaps even more disappointingly, clinical studies evaluating combinations of mTOR and EGFR inhibitors in patients with recurrent glioma demonstrated limited responses and these were not sustained [64], [65]. Thus there is a great deal of preclinical evidence supporting the use of drugs targeting mTOR and EGFR (alone and in combination) but the benefits are not yet being realized in patients.

Recent studies have shed light on the complex protein interactions involving mTOR and these data help explain why mTOR inhibitors were not effective in the clinic. Rictor is part of the mTOR rapamycin-insensitive complex (mTORC2) [18], which functions in a manner that is distinct from mTOR rapamycin-sensitive complex (mTORC1). mTORC2 appears to be essential for the activation of AKT and signaling through mTORC2, promoting cell survival and proliferation [17]. In contrast, inhibition of mTORC1 by rapamycin removes the inhibitory signal of S6K1 on the Insulin Receptor Substrate 1 (IRS1), resulting in the activation of mTORC2 and the PI3K/AKT pathway [11]. Rapamycin treatment increased p(473)-AKT levels in approximately 80% of cell lines tested, suggesting that most cancer cell lines will respond to rapamycin-induced mTORC1 inhibition by activating mTORC2 through this S6K1-mediated feedback loop [11]. These data have led to the suggestion that all non-mTORC2-specific mTOR inhibitors may induce AKT activation while also promoting autophagy [66] and cell survival [19] through mTORC1 inhibition and this, in turn, would affect the therapeutic potential of mTORC2 inhibition. However, little is known about the structure of mTORC2 and how it is regulated by Rictor, and there are no small molecule inhibitors specific to this complex. The studies described in this report have used RNAi-mediated gene silencing methods to assess the consequences of Rictor and EGFR silencing in models of GBM. This study reports for the first time, to our knowledge, that combined suppression of Rictor and EGFR in *in vitro* and *in vivo* GBM models can provide significant therapeutic benefits. There are two important discussion points considered below. The first concerns the challenges of validating a RNAi-mediated targeted therapy approach in orthotopic models of glioma and the second concerns the surprising results suggesting that shRNA-mediated silencing of both Rictor and EGFR resulted in a complete eradication of tumors.

siRNA-transfected-GBM lines were used to assess *in vitro* the therapeutic potential of EGFR and Rictor silencing alone and in combination. Comparisons between the effect of Rictor and ILK siRNA-mediated silencing on AKT phosphorylation (p473) completed in PTEN-mutant U251MG cells confirmed the important role of Rictor in the modulation of (p473)-AKT. The correlation between Rictor silencing and reductions in (p473)-AKT was also confirmed in the PTEN-mutant line U118MG cells, but could not be demonstrated in the PTEN wild-type LN229 cells. It is possible that (p473)-AKT in LN229 cell line relies on other kinases such as ILK [37]–[39], DNA-PK [67], PKCα [68], PDK1 [69], or AKT itself [70]. Other consequences associated with siRNA-mediated silencing of Rictor and/or EGFR included a reduction in cell migration (for U251MG and LN229 cells, Figure 4) and an increase in cell sensitivity to irinotecan, vincristine and temozolomide (Figure 5). It should be noted that previous studies have shown that Rictor can regulate PKC-α and MAPK activity [18], [20], [71]–[73], and it is possible that the effects observed following Rictor silencing in LN229 were the result of inhibition of these pathways rather than through suppression of (p473)-AKT. In the PTEN-negative U118MG, Rictor siRNA alone caused an increase in cell sensitivity to vincristine and temozolomide. The combination of EGFR and Rictor silencing did not provide any additional therapeutic benefits when combined with the selected chemotherapeutics. Results from these *in vitro* studies provided sufficient justification for initiation of the *in vivo* studies.

It is recognized, however, that the use of siRNA therapeutics in GBM remains a challenge in the context of delivery to the target cell population which resides behind the blood-brain barrier. For this reason, the *in vivo* studies focused on use of an inducible shRNA expression system. This system allowed for the characterization of the shRNA transduced lines in the absence of shRNA expression in order to confirm that the lentiviral transduction and subsequent cell selection did not alter the characteristics of the cell population. Indeed, assessments *in vitro* (MTT proliferation assay) and *in vivo* (s.c. inoculation) of transduced tumor cell growth rates confirmed that the cell lines generated behaved comparably to each other and to the parental line in the absence of doxycycline. Furthermore, the *in vitro* sensitivity of these cells to selected drugs in the absence of doxycycline was also comparable. The utility of this system for validating silencing effects *in vivo* is further highlighted by the tRFP expression data shown in Figure 8. The doxycycline-induced expression of shRNA in each cell line and in tumors could be directly confirmed using fluorescence microscopy and non-invasive imaging.

Following doxycycline induction, shRNA-mediated silencing of EGFR or Rictor alone was shown to have a small and insignificant impact on U251MG tumor development. Surprisingly, three independent studies demonstrated that doxycycline induction in animals inoculated with U251MG cell line expressing shRNAs targeting both Rictor and EGFR resulted in a complete eradication of tumors. This synthetic lethal effect was not expected based on the *in vitro* studies completed with siRNA.

The fact that the combined silencing of EGFR and Rictor led to tumor eradication may have been anticipated on the basis of previous publications which have demonstrated the involvement of both proteins in tumor progression. EGFR signaling involves the PI3K/AKT, STAT3, MAPK and BCL-X_L_ pathways regulating apoptosis, proliferation and differentiation (reviewed in [25]). The mTORC2/Rictor complex was shown to regulate apoptosis, cell cycle and invasion through AKT and PKC-α pathways [18], [20]. Interestingly, our results showed that suppression of EGFR or Rictor alone did not significantly affect tumor growth *in vivo*, and had little impact on cell proliferation, apoptosis and migration *in vitro*. siRNA-mediated knockdown of Rictor was also previously reported to have no effect on cell proliferation, survival and invasion [71]. The absence of significant therapeutic effects associated with EGFR inhibition described here also correlates with results observed in the clinic [61], [74]–[76] using small molecule EGFR inhibitors, and in some pre-clinical GBM studies [77]–[79] using small molecule EGFR inhibitors or siRNA. More specifically, Fan *et al* reported that the EGFR inhibitor gefitinib or the pan-PI3K inhibitor LY294002 given as monotherapy had no impact on tumor burden in a GBM model, yet when these inhibitors were used in combination, they blocked tumor growth [78]. Synergistic effects between mTOR and EGFR inhibitors in GBM have also been reported elsewhere [79], [80]. Taken together, the synthetic lethality observed in the studies reported here are consistent with previously published reports and support the use of EGFR and Rictor pathway suppressors to achieve optimal therapeutic effects that may not be observed by inhibition of either pathway alone.

It should be noted, however, that the published literature is not entirely consistent when considering the effects of targeting these proteins. Reports have shown that GBM cells transduced with antisense oligonucleotide or shRNA expression plasmids specific to EGFR exhibit increased apoptosis, cell cycle arrest and reduced cell proliferation rate *in vitro* [81]–[83]. Similarly, previously reported studies have suggested that GBM cells expressing shRNA specific to Rictor exhibited reduced cell proliferation and migration *in vitro* and inhibition in tumor growth *in vivo* [20]. The expression vectors, transfection/transduction methods and shRNA sequences used in these reports were different from those used here, however, and it is possible that these systems have generated different levels of target knockdown or have affected signaling and phenotype differently when compared to the methodology described in this report.

The observed synergy between Rictor and EGFR silencing in U251MG and LN229 cells could be explained by possible inhibition of compensating pathways induced by silencing of each targets. For example, resistance to EGFR inhibition in GBM was previously suggested to be due to activation of IGF1R [84] or MET [85] pathways, and signaling from these pathways results in AKT activation [84], [86], [87]. Silencing of Rictor in combination with EGFR silencing may thus prevent activation of AKT in response to EGFR inhibition. On the other hand, Rictor silencing was shown to activate Raf-1-MEK-ERK pathway in glioma cells [71]. The Raf-1-MEK-ERK pathway is also modulated by EGFR [25], and it is possible that silencing of EGFR in combination with Rictor inhibition may decrease the level of activation of Raf-1-MEK-ERK in response to Rictor silencing. Therefore, combined silencing of EGFR and Rictor may result in increased and more stable inhibition of pathways regulating cellular functions involved in malignancy. More specifically, the reduction in cell motility induced by the combined silencing of EGFR and Rictor described in this report may involve inhibition of pathways regulated by both targets, such as PKC and AKT [25], [45], [48]–[52], [88]. In addition, EGFR and Rictor combined silencing may have promoted a greater shift towards apoptosis in response to the chemotherapeutic agents tested in this report, as both EGFR and Rictor are involved in pro-survival and DNA repair pathways [89]–[92]. Other assays assessing cell invasion and adhesion capability, as well as radiosensitivity and anchorage-independent growth in EGFR and Rictor downregulated cells will be interesting to perform as part of future studies aimed at further documenting the therapeutic potential of the combined silencing approach. The effects of the combined silencing of EGFR and Rictor in other GBM cells expressing different levels of EGFR or Rictor will need to be done to confirm the observations noted here.

No methods for specific and efficient delivery of siRNA therapeutics to GBM tumors are currently available, and the dual targeted therapy validated here using RNAi-mediated gene silencing methods is not likely to be applicable to the clinic immediately. However, considerable research effort has been invested in this field and it can be expected that a viable siRNA delivery option will become available in a short future. Thus, the present study consists of a pre-clinical validation of the therapeutic potential of the combined inhibition of EGFR and Rictor. The *in vitro* data suggest that EGFR and Rictor dual suppression increased tumor cell sensitivity to temozolomide, irinotecan and vincristine; drugs which have been proven to prolong the survival time of GBM patients. A treatment including the combined suppression of the targets together with chemotherapy or radiation should then be considered. Most importantly, the *in vivo* data suggests that the dual inhibition of EGFR and Rictor produce significant inhibition of tumor growth, even in the absence of chemotherapy. The concept that there is a pool of dormant brain tumor initiating cells (BTIC) existing within a tumor has emerged over the last few years, and these cells have been described as highly invasive and resistant to chemotherapeutic agents that target actively proliferating cells [93]. It has been proposed that after therapy or surgery, these cells are capable of entering the cell cycle to replenish the tumor cell population [94]. The fact that dual silencing of EGFR and Rictor led to tumor eradication may suggest that such treatment targeted the BTICs. Obviously, additional studies are needed to address these important questions and will include a comprehensive assessment of tumor phenotype immediately after doxycycline administration, as well as an assessment of the fate of the BTIC population (CD133+ cells) in the *in vivo* model used here. Moreover, studies aimed at defining a delivery strategy for siRNA in GBM tumors are currently ongoing in our laboratory, and the strategy tested may allow the assessment of therapeutic siRNAs specific to EGFR and Rictor in GBM in the near future.
